# Accuracy of Broselow tape in estimating the weight of the child for management of pediatric emergencies in Nepalese population

**DOI:** 10.1186/s12245-020-0269-0

**Published:** 2020-02-12

**Authors:** Pukar K.C., Akhilendra Jha, Kamal Ghimire, Roshana Shrestha, Anmol Purna Shrestha

**Affiliations:** 1 School of Medical Sciences, Kathmandu University, Kavrepalanchowk, Nepal; 2 Emergency Department, Dhulikhel Hospital, Kathmandu University Teaching Hospital, Kavrepalanchowk, Nepal

**Keywords:** Broselow tape, Resuscitation, Pediatrics, Endotracheal tube, Adrenaline

## Abstract

**Background:**

Children with emergency conditions require immediate life-saving intervention and resuscitation. Unlike adults, the pediatric emergency drug dose, equipment sizes, and defibrillation energy doses are calculated based on the weight of the individual child. Broselow tape is a color-coded length-based tape that utilizes height/weight correlations for children. However, in low-income countries like Nepal, due to factors like undernutrition, the Broselow tape may not accurately estimate weight in all ranges of pediatric age group.

**Methods:**

This study was conducted in the Department of Pediatrics of Dhulikhel Hospital, Kathmandu University Teaching Hospital, in children less than 15 years of age. Our study aims to prospectively compare the actual weights of urban and rural Nepalese children with the estimated weights using the Broselow tape (2017 edition) and the updated APLS formula. The errors in the selection of endotracheal tube size and adrenaline dose using the Broselow tape were also explored.

**Results:**

This study included 315 children with male to female ratio of 0.63:1. They were divided into 3 groups according to their estimated weight by the Broselow tape into < 10 kg, 10–18, and > 18 kg. There was a total agreement of the estimated color zone according to the Broselow tape with the actual weight in the gray zone (*p* = 0.01). There was a positive relationship between the actual body weight and the estimated body weight (correlation (*r* = 0.970, *p* = 0.01) and accuracy (*r*^2^ = 0.941)). Our analysis showed that the accuracy of estimated weight with the Broselow tape decreases with increasing weight of children. The precision of the tape was relatively high in the lower length zones as compared to the higher length zones. The estimated size of the endotracheal tube (*p* = 0.01) and adrenaline dose (*p* = 0.08) by the Broselow tape was in agreement with that estimated using PALS formula in weight group of less than 18 kg, but decreases as the estimated weight increases further.

**Conclusions:**

The accuracy of the Broselow tape in estimating the weight of a child, endotracheal tube size, and dose of adrenaline is higher in weight group of less than 18 kg, and accuracy decreases as the weight of child increases. The Broselow tape should be avoided in children weighing more than 18 kg. Hence, PALS age-based formula for ET tube size estimation and weight-based formula for adrenaline dose calculation are recommended for children weighing more than 18 kg.

## Background

Children with a wide variety of urgent medical and surgical conditions visit the emergency department (ED), which requires immediate life-saving intervention and resuscitation. The accurate measurement or estimation of the weight of a child is crucial for the effective and optimal acute management of pediatric emergencies. In contrast to adults, the pediatric emergency drug dose, equipment sizes, and defibrillation energy doses are calculated based on the weight of the individual child and are a challenge for the treating emergency physician. The medical error related to the calculation of pediatric medication dosage is very high [1]. Incorrect estimation of patient weight, leading to incorrect drug dosing, is one of the most frequently reported errors [2]. It is not always feasible to measure the weight of a child using the standard weighing machine in the ED where the condition is critical and immediate action is required. When the accurate weight of the child cannot be obtained, it is usually calculated using an age-based formula such as advanced pediatric life support (APLS) formula which can be incorrect and time-consuming [3]. Wrong estimation of weight or incorrect calculation of drug dosage or equipment size could result in grave consequences.

Length-based weight estimation is developed as a different alternative to estimate the weight. The Broselow pediatric emergency tape is a color-coded length-based tape measure that was developed using height/weight correlations for children who have a maximum weight of roughly 36 kg from a nationally representative sample of children in the USA [4, 5]. The Broselow tape also provides medical instructions including the medication dosages, the size of the equipment, and the level of shock voltage when using a defibrillator. It is recognized in most medical textbooks and publications as a standard for the emergency treatment of children and is recommended by the Advanced Trauma Life Support and Pediatric Advanced Life Support [4, 6]. In a critical life-threatening condition, it is not judicious to consume the valuable time needed to evaluate, initiate, and monitor patient treatment to calculate the estimated weight, the equipment sizes, and the drug doses. The pre-calculated dosing in the Broselow tape facilitates rapid weight estimation, saves time by providing corresponding drug dosing, and alleviates stress during pediatric resuscitation [5, 7–9]. In a simulated pediatric emergency, color coding significantly reduced the deviation from recommended doses [8]. The studies done in various parts of the world have shown that the Broselow tape accurately measured the weight of children [10–12] while some studies showed it to be inaccurate [13–17]. The scenario is quite different in low-income countries like Nepal [15, 18, 19]. The undernutrition is still a major concern among pediatrics population in Nepal [20]. So, the recent modifications made given of obesity prevalence in western society do not address the issues of developing countries, which may lead to substantial overestimation of the weight of the children and potentially dangerous drug dosing and equipment selection [21]. A study done in urban Nepal by Shrestha et al. showed that the Broselow tape (2007 B edition) had only moderate accuracy for weight estimation [22].

This study aimed to prospectively compare the actual weights of urban and rural Nepali children with the estimated weights using the Broselow tape (2017 edition) and the updated APLS formula. The errors in the selection of endotracheal tube (ET) size and adrenaline dose using the Broselow tape were also explored.

## Methods

### Study design

This was a prospective cross-sectional study conducted from March to June 2019.

### Study setting

The study was conducted in the Department of Pediatrics (Outpatient and Immunization Section) of Dhulikhel Hospital, Kathmandu University Teaching Hospital, Kavrepalanchowk. The hospital is a tertiary care referral center that provides health care services to a population of approximately 1.9 million people from the urban and rural area of Kavrepalanchowk, Sindhupalchowk, Dolakha, Sindhuli, Ramechhap, Bhaktapur, and other surrounding districts.

### Participants

All the children visiting the department under 15 years of age were eligible for the study.

Exclusion criteria were set for convenience:

A. Children with height outside of the Broselow tape’s limit (less than 46.5 cm and above 142.5 cm)
B. Children with chronic disease and who were under medications for prolonged duration; outcome may be compromised if readings of the abovementioned categories were taken into account as the height and weight may be different from the general population.
C. Children with medical emergencies who require immediate medical intervention; it would have inappropriately delayed in receiving the emergency care during busy hours of the emergency department.

The Broselow pediatric emergency tape (Armstrong Medical Industries, Lincolnshire, IL, USA, 2017 edition) available in the ED of the hospital was used for this study purpose. The Broselow tape has 9 colored zones. For the study purpose, the color zones were further grouped into 3 groups with 3 color zones into each group, viz, group 1 (< 10 kg)—grey, pink, and red; group 2 (10–18 kg)—purple, yellow, and white; and group 3 (> 18 kg)—blue, orange, and green. A sample of 96 participants was required in each group to detect a 10% difference between actual weight and the Broselow estimated weight with an aggregate sample size of 288 using the sample calculation for standard normal deviate assuming 50% as the proportion of the target population.

Demographic data was collected from the guardian using a pre-designed questionnaire after informed consent was obtained. The weight of the candidates was measured by standard calibrated weighing machine to the nearest 0.1 kg using an electronic weighing machine. The shoes and heavy clothing were removed before measuring the weight. Another investigator was blinded to the age, measured the weight of the child, and recorded the estimated weight of the child using the Broselow tape in supine position measured from head to heel. Using the age provided by the caregiver, the child’s weight was calculated using the updated APLS formulae—weight (kg): infant 0–12 months = (0.5 × age in months) + 4; children aged 1–5 years = 2 × age (years) + 8; 6–12 years = 3 × age (years) + 7.

The size of the internal diameter (ID) of the ET tube was calculated using Pediatric Advanced Life Support (PALS) recommendations: 3.5-mm ID uncuffed ET tube for infants up to 1 year of age and 4.0-mm ID uncuffed ET tube for children between 1 and 2 years of age. After age of 2, by the following formula: age/4 + 4 for uncuffed and age/4 + 3.5 for cuffed ET tube [6]. The size of the calculated ET tube was rounded off reducing to the nearest 0.5 mm ID. The calculated size was compared with the size recommended according to the corresponding Broselow zone. The calculated dose of adrenaline (0.01 mg/kg 1:10,000) was compared with the estimated dose according to the Broselow tape. ET tubes with an ID 0.5 mm smaller or larger than the estimated size are recommended to be made available during the intubation attempt. Therefore, an acceptable ET tube size was defined as the ET tube size ranging within 0.5 mm size of the calculated size. The acceptable dose of adrenaline was defined as a drug dose within the range of ± 20% from the recommended dose [9]. Any values beyond these recommendations were considered to be an error.

## Data analysis

The categorical variables were presented by frequency and percentage, and the continuous variables by mean with standard deviation and range. The percentage of agreement and over- or underestimation within the one/two color zone of the estimated color zone according to the Broselow tape with the actual weight was calculated. The relationship between the actual body weight and the estimated body weight was analyzed by Pearson’s correlation test. The Bland-Altman analysis was performed to evaluate the measures of bias, precision, and accuracy [23]. The mean percentage error (MPE = mean of the sum of [estimated weight − actual weight/actual weight] × 100) limits of agreement (LOA) was used to evaluate the bias and precision, whereas the percentage of weight estimations within 10% and 20% of actual weight (PW10 and PW20) was calculated to evaluate the accuracy of the Broselow tape and the updated APLS formula. All the analysis was carried out using SPSS version 21. A *p* value less than 0.05 was considered significant.

## Results

This study included 315 children with male to female ratio of 0.63:1. They were divided into 3 groups according to their estimated weight by the Broselow tape into < 10 (gray, pink, and red color code), 10–18 (purple, yellow, and white color code), and > 18 kg (blue, orange, and green color code) which comprised 100, 113, and 102 children, respectively. The demographic and descriptive details of the enrolled children are illustrated in Table 1.

**Table 1:** Demographics of the children

Parameter	Weight group			
	Group 1, < 10 kg (*n* = 100)	Group 2, 10–18 kg (*n* = 113)	Group 3, > 18 kg (*n* = 102)	Total (*n* = 315)
Mean age (months) ± SD (minimum–maximum)	5.15 ± 3.69 (0–16)	30.80 ± 16.77 (5–78)	102.39 ± 30.13 (48–164)	45.92 ± 45.17 (0–164)
Measured weight (kg), mean ± SD (minimum–maximum)	6.42 ± 1.83 (3.1–10)	11.86 ± 2.44 (7–18)	24.04 ± 5.34 (14–36.1)	14.09 ± 8.05 (3.1–36.1)
Estimated weight by the Broselow tape (kg), mean ± SD (minimum–maximum)	6.55 ± 1.79 (3–9)	13 ± 2.56 (10–18)	27.07 ± 5.88 (19–36)	15.53 ± 9.25 (3–36)
Female, *n* (%)	39 (39.4)	41 (36)	42 (41.2)	122 (38.7)

*n* number of cases, *SD* standard deviation

The agreement of the estimated color zone according to the Broselow tape with the actual weight is demonstrated in Table 2. A total agreement was seen in the gray color zone, and the convergence gradually decreased with increasing length and weight of the children (*p* = 0.01).

**Table 2:** Agreement between estimated color zone by the Broselow tape and the actual weight

Estimated color zone according to the Broselow tape	Color zone agreement according to actual weight, *n* (%)			Total (*n*)
	Agreement on color zone	1 color zone difference	2 color zone difference	
Gray	33 (100)	0	0	33
Pink	25 (80.6)	6 (19.3)	0	31
Red	21 (58.3)	15 (41.7)	0	36
Group 1 (< 10 kg)	**79 (79)**	**21 (21)**	**0**	**100**
Purple	14 (35.9)	22 (56.7)	3 (7.7)	39
Yellow	18 (46.2)	20 (51.3)	1 (2.6)	39
White	18 (51.4)	15 (42.9)	2 (5.7)	35
Group 2 (10–18 kg)	**50 (44.3)**	**57 (50.4)**	**6 (5.3)**	**113**
Blue	16 (45.7)	18 (51.4)	1 (2.9)	35
Orange	11 (44)	14 (56)	0	25
Green	15 (35.7)	21 (50)	6 (14.3)	42
Group 3 (> 18 kg)	**42 (41.2)**	**53 (51.9)**	**7 (6.9)**	**102**
Total	**171 (54.3)**	**131 (41.6)**	**13 (4.1)**	**315**

**Fig. 1:**
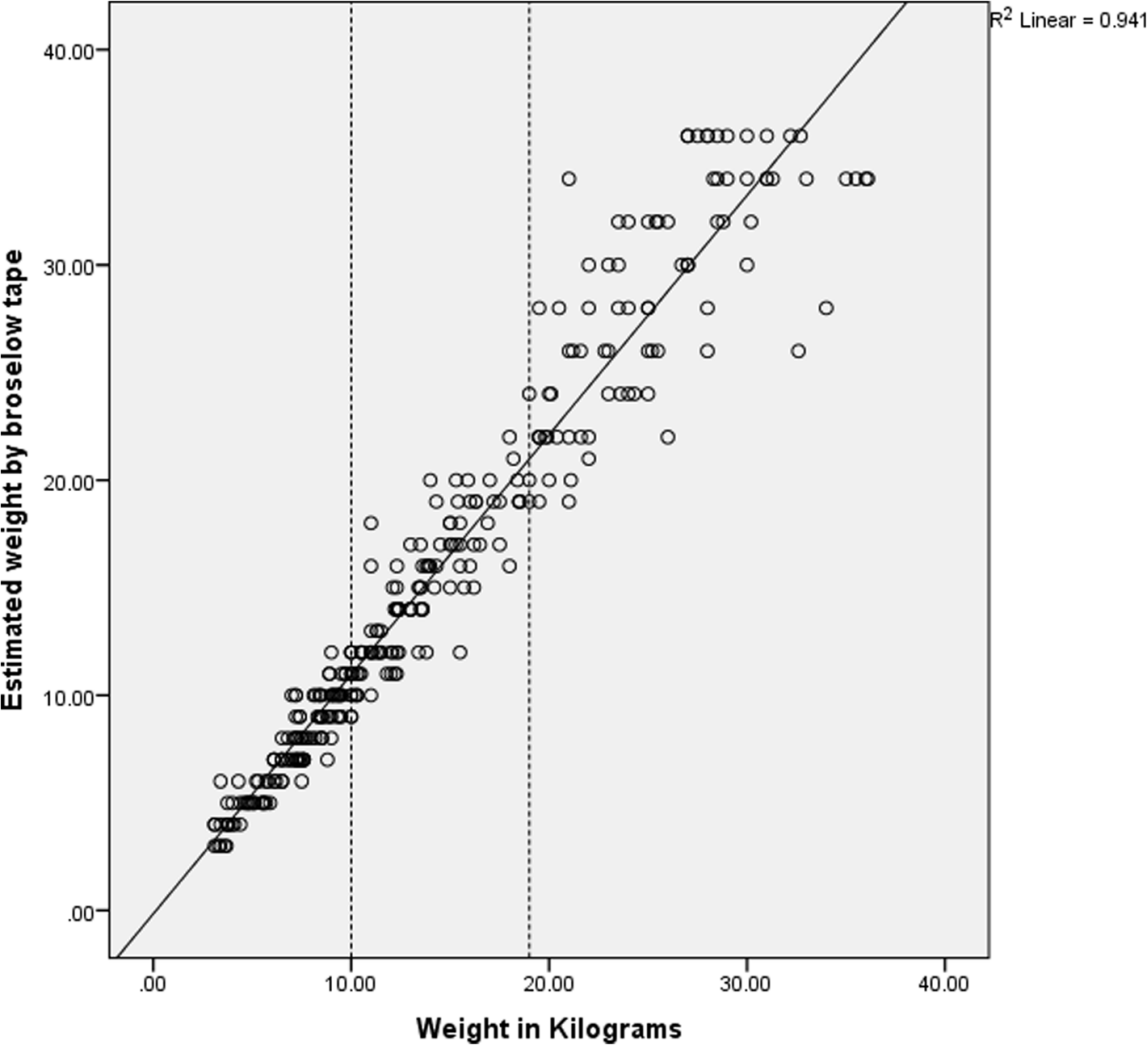
Correlation between the measured weight and the Broselow tape estimated weight. Correlation (*r* = 0.970, *p* = 0.01) and accuracy (*r*^2^ = 0.941)

The relationship between the actual body weight and the estimated body weight was analyzed which demonstrated a positive relationship (Fig. 1) (correlation (*r* = 0.970, *p* = 0.01) and accuracy (*r*^2^ = 0.941)). The crowding of data points is noticed below 10 kg and starts to scatter more as the weight of the child increases. This indicates that the accuracy of estimated weight with the Broselow tape decreases with increasing weight of children.

**Fig. 2:**
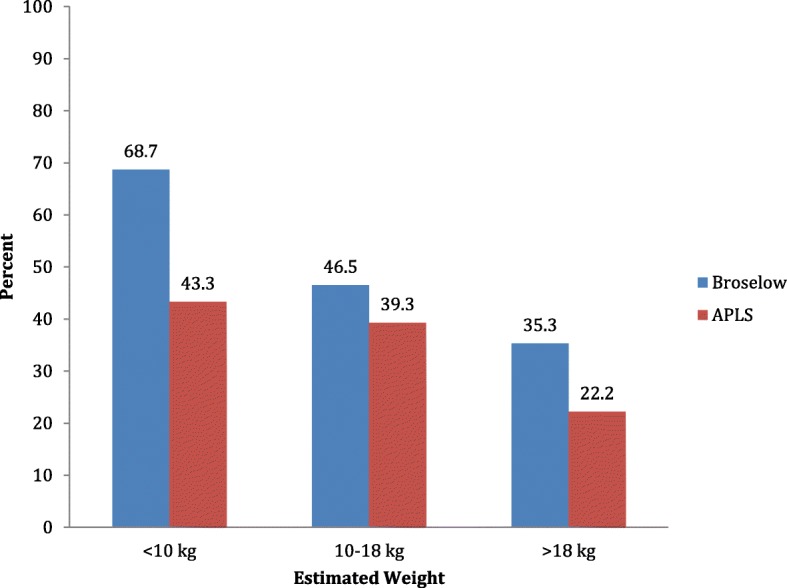
Comparison of PW 10% between weights estimated by the Broselow tape and modified APLS formula. Comparison of percent within 10% (PW10) of actual weight measured by weighing machine to that estimated by the Broselow tape and updated APLS formula

The measures of bias and accuracy for the Broselow tape and APLS formula are shown in Table 3. The mean percentage error (MPE) with the Broselow tape was lowest for the < 10 kg (3.11%) and gradually increased in the 10–18 kg (10.57%) and > 18 kg (13.55%) groups. The Broselow tape demonstrated the similar bias of weight estimation compared to the APLS formula in the first two groups, except in the > 18 kg group, where the bias was more in APLS formula weight estimation group (13.55 versus 32.60). The predicted weight within the PW10 was highest among the < 10 kg group for both the Broselow tape and APLS formula (68.7% and 43.3%, respectively) and lowest among the > 18 kg group (35.3% and 20%, respectively) (Fig. 2).

**Table 3:** Measures of bias, precision, and accuracy for the Broselow tape and APLS formula for weight estimation

Parameters	Broselow tape	APLS
< 10 kg		
MPE ± SD (maximum–minimum)	3.11 ± 13.87 (− 20.45–76.47)	3.1359 ± 16.88 (− 30–45.16)
PW10, *n* (%)	68 (68.7)	45 (43.3)
PW20, *n* (%)	88 (88.9)	79 (76)
10–18 kg		
MPE ± SD (maximum–minimum)	10.57 ± 12.53 (− 22.6–63.6)	10.41 ± 14.03 (− 23.26–51.59)
PW10, *n* (%)	53 (46.5)	42 (39.3)
PW20, *n* (%)	98 (86)	79 (73.9)
> 18 kg		
MPE ± SD (maximum–minimum)	13.55 ± 14.14 (− 20.25–61.9)	32.60 ± 25.53 (− 16.60–102.65)
PW10, *n* (%)	36 (35.3)	18 (20)
PW20, *n* (%)	70 (68.6)	33 (26.7)
All		
MPE ± SD (maximum–minimum)	9.19 ± 14.12 (− 22.58–76.47)	14.53 ± 22.58 (− 30–102.65)
PW10, *n* (%)	157 (49.8)	105 (34.9)
PW20, *n* (%)	256 (81.2)	191 (63.5)

Mean percent error (MPE): [(measured weight − Broselow estimated weight)/actual weight] × 100

*PW10* percent within 10%, *PW20* percent within 20%

**Fig. 3:**
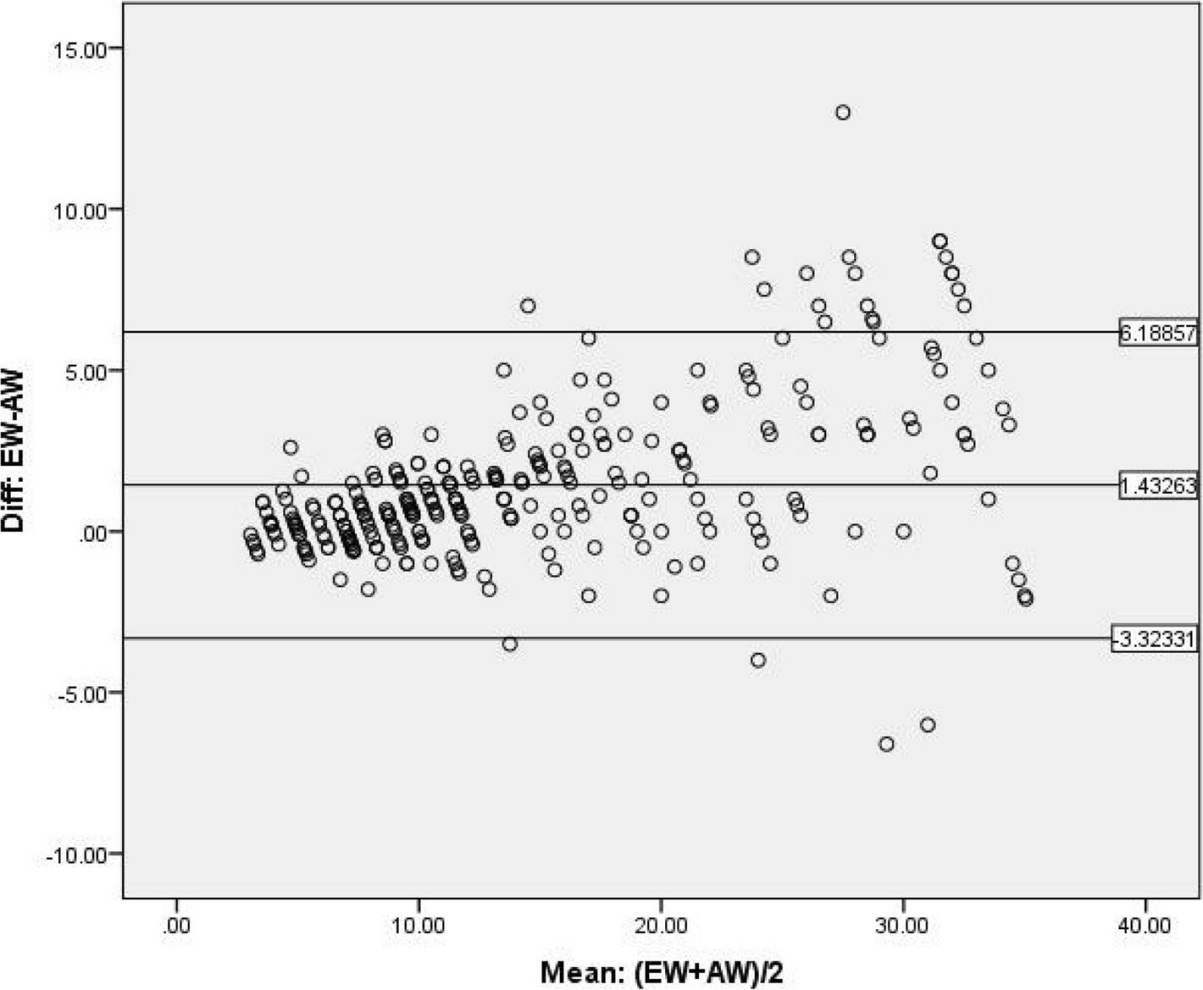
The Bland-Altman plot of differences between actual weight and estimated weight by the Broselow tape. EW, estimated weight by the Broselow tape; AW, actual weight measured by weighing machine; upper limit (UL), 6.18857; lower limit (LL), − 3.32331; mean, 1.43263; confidence interval (CI), 95%

A Bland-Altman plot was drawn to evaluate the agreement between the actual weight and the MPE (Fig. 3). The upper and lower Bland-Altman limits of agreement were 6.18857 and − 3.32331, respectively, with a confidence interval of 95%. The plot demonstrates that the difference was scattered as the weight of the children increased; therefore, the precision of the tape was relatively high in the lower length zones as compared to the higher length zones. The Broselow tape estimated ET tube size was within the PALS recommendation and acceptable limit of within 0.5 size of recommended size in 88.76%, 94.9% in weight less than 10 kg, and 93.9% in weight 10–18 kg. Adrenaline drug dose estimation in weight group of < 18 kg is in accordance with actual weight-based calculation of drug dosing, showing 75.8% and 74.6% accuracy for weight group < 10 kg and 10–18 kg, respectively, which fails to predict the dose accurately as the weight increases, showing 64.7% for weight group of > 18 kg with acceptability limit of + 20% (Table 4).

**Table 4:** ET tube size and adrenaline dose accuracy for estimated weight according to the Broselow tape

Weight group	Recommended ET tube size ID, *n* (%)			Recommended adrenaline dose, *n* (%)		
	± 0.5 mm	< 0.5 mm	> 0.5 mm	± 20%	< 20%	> 20%
< 10 kg	94 (94.9)	5 (5.1)	0	75 (75.8)	3 (3)	21 (21.2)
10–18 kg	107 (93.9)	6 (5.3)	1 (0.9)	85 (74.6)	0	29 (25.4)
> 18 kg	79 (77.5)	6 (5.9)	17 (16.7)	66 (64.7)	1 (1)	35 (34.3)
Total	280 (88.76)	17 (5.43)	18 (5.86)	226 (71.7)	4 (1.3)	85 (26.96)

Acceptable ET tube size was defined as the ET tube size ranging within 0.5 mm size of the calculated size. The acceptable dose of adrenaline was defined as a drug dose within the range of ± 20% from the recommended dose

## Discussion

This study explored the bias, precision, and accuracy of the Broselow tape in the prediction of the weight of children visiting the outpatient pediatric department and vaccination unit of a tertiary care hospital which serves the population from urban and rural areas of Nepal. The agreement of the estimated color zone according to the Broselow tape with the actual weight was closest in group 1 (< 10 kg) and gradually decreased with increasing length and weight of the children. The relationship between the actual body weight and the estimated body weight was analyzed which indicated that the accuracy of estimated weight with the Broselow tape decreases with increasing weight of children.

For pediatric resuscitation, precise weight estimation is critical as the emergency drugs, equipment, and shock dosage are based on the weight of the individual child. We used the 2017 version of the Broselow tape for this study which was available in the ED. A reference at each color zone on the tape suggests equipment sizes to perform emergency resuscitation and pre-calculated medication dosages. Pre-calculated doses in milligrams, as well as all doses in pre-calculated ml in the 2017 version, remove the need for further calculations and may decrease the error related to the calculation. The Broselow cart with 9 color-specific drawers which matched the colors on the Broselow tape was constructed locally and utilized in our ED for emergency pediatric care. With this method, the drug dosage and equipment sizing could easily and quickly be determined and accessed. However, it was a concern of inaccuracy in LMIC due to underweight, lack of research in the field. Moreover, it is a concern that the newer version of the tape is more inaccurate than older versions of the tape in low- and middle-income countries because its shifted weight zones due to raised concerns regarding underestimations of drug dosages secondary to obesity in high-income country growth data [24]. Using newer edition of the tape presumably creates a greater error in estimating the weight of our populations compared to prior edition as it has been modified to minimize weight underestimation in obese children in the USA.

There are studies done in various parts of the world providing the adaptability and efficiency of the Broselow tape in measuring the weight of children [10, 11, 25] while other studies had contradictory conclusions [13–17, 22]. Studies from Kenya [10] and South Africa [10, 25] showed that the Broselow tape estimated weights varied only minimally from the actual weight. Clark et al. discussed their concern about using the Broselow tape for weight estimation among children with malnutrition in low-income countries like Sudan. They concluded that both the Broselow tape and age-based formulas were markedly inaccurate in both the non-malnourished and malnourished African children. Moreover, it was unacceptable in malnourished children and they suggested further studies to explore appropriate methods of weight and dose estimation for children from regions with a high prevalence of malnutrition.

A study done in India also showed similar results to ours that the results were favorable in the children < 10 kg group and 10–18 kg group, and they also had the opinion that as pediatric weight increases, the reliability of the tape significantly plummeted [17]. The Broselow tape overestimated weight by > 10% in the majority of Indian children, and they suggested to apply 10% weight correction factor to the Broselow estimated weight [18, 19]. Sinha et al. [26] compared the actual weight with the Broselow estimated weight in pediatric trauma patients and concluded that the bias was greatest in the highest weight category.

The measures of bias and accuracy for the Broselow tape and APLS formula were similar for the < 10 kg and 10–18 kg group. While comparing the estimated Broselow weight against APLS formula, the former met better accuracy especially in children > 18 kg. Another study also demonstrated that weights estimated using the Broselow tape correlated better with actual weights than those calculated using the APLS and updated APLS formulae [19].

A single study which was done in Nepal [22] compared the measured weight with the estimated weight of Broselow, PAWPER XL [27], and Mercy tapes [28]. The study concluded that PAWPER XL tape provides the most accurate weight estimation of children in Nepal followed by the Broselow tape. The accuracy remained high for children weighing over 20 kg, unlike the other two methods. However, the PAWPER tape is not easily accessible in resource-limited settings. When we compare the results of our study with that of this study, the accuracy was lower for the Broselow tape in all weight groups, PW10 (49.8 versus 63.2) and PW20 (81.2 versus 91.7). This could be due to the different patient characteristics and use of the newer version of tape in our study.

The overestimation of equipment size and the drug doses may lead to serious side effects and delay in resuscitation. A study conducted in India showed the accuracy of the Broselow tape in estimating the ET tube size was 50.3% in children less than 6.5 years of age and weight of less than 15 kg [29]. We tested the equipment size (ET tube) and drug dose (adrenaline) to check for the accuracy of their estimation using the Broselow tape. The ET tube size and Adrenaline dose estimation by using Broselow Tape was within acceptable limit for weight group <10 kg and 10-18 kg.

Based on the findings of this study, we can demonstrate that the Broselow tape can be safely used in children weighing less than 18 kg and should be avoided in children weighing more than 18 kg. This finding contradicts our research hypothesis; the Broselow tape overestimates the weight, equipment size, and drug doses in pediatric age group; and drug overdose would affect the children with smaller weight. However, the study showed the Broselow tape to be more accurate in estimating the weight, equipment size, and drug doses in children with weight less than 18 kg.

## Limitation

The study was conducted at a single hospital and cannot be generalized to the whole Nepalese population. Only the children attending the hospital are included in the study, not the general population which may not fulfill the true purpose of the study. Our study compared only limited drug dose and equipment size, but the Broselow tape recommends multiple other drug doses and equipment sizes. Our study could not provide recommendations for estimating weight of children in age of more than 18 kg, so further study is required in the field.

## Conclusions

The Broselow tape is more reliable in children of the < 10 kg and 10–18 kg groups. However, as pediatric weight is increased, predictive reliability is also decreased, especially in those weighing > 18 kg. However, the Broselow tape was found to be superior to updated APLS formula in estimating the weight of child in all weight groups.

Thus, medical errors will be reduced by the implementation of the Broselow tape in Nepal to estimate pediatric weight accurately in the emergency setting for children of weight group < 18 kg, but an alternative method for weight estimation is suggested for weight group > 18 kg in Nepal.

Similarly, the estimation of ET tube size and adrenaline drug dose by using the Broselow tape is more accurate and reliable in children of < 18 kg and its predictive reliability decreases as weight increases. Thus, age-based PALS formula is recommended to be used for estimating ET tube size and weight-based formula for adrenaline dose in children above 18 kg weight group.

## Data Availability

The datasets used and/or analyzed during the current study are available from the corresponding author on reasonable request.

## Abbreviations

APLS: Advanced pediatric life support
ED: Emergency department
ET tube: Endotracheal tube
ID: Internal diameter
PALS: Pediatric Advanced Life Support
